# Effect of excessive internet gaming on inhibitory control based on resting EEG and ERP

**DOI:** 10.1016/j.isci.2024.110399

**Published:** 2024-06-27

**Authors:** Jiayi Xu, Lu Shen, Huajia Fei, Wenbin Zhou, Feng Wan, Wenya Nan

**Affiliations:** 1 School of Psychology, Shanghai Normal University, Shanghai, China; 2 Department of Electrical and Computer Engineering, Faculty of Science and Technology, University of Macau, Macau, China; 3 Centre for Cognitive and Brain Sciences, Institute of Collaborative Innovation, University of Macau, Macau, China

**Keywords:** Behavioral neuroscience, Clinical neuroscience, Social sciences

## Abstract

Previous research indicates that individuals with Internet gaming disorder (IGD) show impaired inhibitory control and abnormal EEG/ERP patterns. However, it is unclear how those individuals with excessive Internet game use (EUG) but without addiction differ. This study examined inhibitory control, resting EEG, and ERP in EUG gamers compared to non-gamers. Fifteen participants in each group underwent 4-min eyes-closed EEG recordings and a color-word Stroop task. Results showed no significant differences in reaction time, accuracy, or P3 amplitude between EUG gamers and non-gamers. However, EUG gamers exhibited shortened P3 latency, which may suggest enhanced inhibitory control. Additionally, EUG gamers showed reduced theta and alpha band power during the resting state compared to non-gamers. These findings suggest that excessive gaming without addiction may enhance inhibitory control and influence brain activity differently from IGD.

## Highlights

• Inhibitory control behaviors show no difference between EUG gamers and non-gamers
• EUG gamers exhibited shortened P3 latency compared to non-gamers
• EUG gamers showed reduced resting theta and alpha power compared to non-gamers

## Graphical abstract

**Figure:**
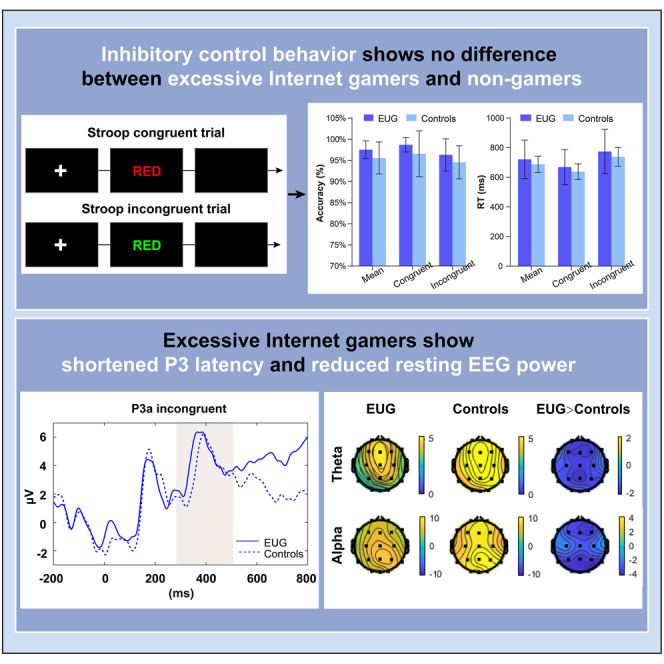

## Introduction

According to the China Internet Network Information Center (CNNIC), the number of Internet users in China has reached 1.079 billion as of June 2023, with Internet penetration being 76.4%, and the population of online gamers in China has increased 11.09 million since December 2022. As playing online games has become a more and more popular activity among adolescents and adults, its impact on public health has attracted researchers' attention worldwide.^1^

Internet gaming disorder (IGD) in the appendix of the Diagnostic and Statistical Manual of Mental Disorders (5^th^ ed., DSM-5) is described as “persistent and recurrent use of Internet to engage in games, often with other players, leading to impairment or clinically significant distress.” Moreover, according to the 11th Revision of the International Classification of Diseases (ICD-11), IGD has been classified as a type of mental illness and regarded as behavioral addiction or impulse control disorder.^2^ It has also become a common mental health problem among Asian teenagers. For example, the prevalence of IGD in 12–26 years students is 10.3% in China.^3^

As one of the core cognitive control functions, inhibitory control represents the ability to control one’s attention, thoughts, emotions, and behaviors to inhibit desires and do what is more appropriate or needed instead.^4^ Inhibitory control is negatively related to impulsivity.^5^ High impulsivity, or called impaired inhibitory control, may increase one’s vulnerability to developing addictive disorders.^6^

Many neuropsychological studies have demonstrated impaired inhibitory control and related abnormal brain activities in IGD.^7^^,^^8^^,^^9^^,^^10^^,^^11^ For instance, compared with non-gamers, participants with IGD showed a deficiency in response, working memory, and processes related to inhibitory control.^10^ Prior studies have also found abnormal resting EEG activities in the IGD population. More specifically, IGD individuals exhibit increased delta and theta power,^12^^,^^13^^,^^14^ reduced beta power,^15^^,^^16^ and enhanced gamma power with eyes closed.^15^ Among the EEG rhythms, both theta and alpha activities have been linked to inhibitory control. Specifically, the resting theta power works as "alarm" signals to process stimulus and recognize the need for inhibitory control, and is considered to be related to response inhibition performance.^17^ The higher theta activity is associated with lower inhibitory control.^18^^,^^19^ Resting-state alpha power has been shown to tie up with attention span, and the decreased alpha power at rest reflects a release from cortical inhibitory control and the requirement for attentional resources.^20^^,^^21^^,^^22^^,^^23^^,^^24^ In addition, the IGD population exhibited abnormal event-related potentials (ERPs). More specifically, compared to healthy controls, the P3 component, i.e., a positive-going wave emerging 300–500 ms after stimulus onset, shows diminished P3 amplitude with an auditory oddball task.^25^

It is worth noting that the above findings are from individuals with IGD. Different from IGD patients, people with excessive use of Internet games (EUG), also called recreational Internet game users or casual gamers, play Internet games excessively or recreationally but without addiction.^26^^,^^27^^,^^28^ Importantly, they do not meet the criteria for IGD by DSM-5. Previous studies have indicated that EUG can lead to a wide variety of negative psychosocial consequences, dysfunctional behaviors, as well as health and medical consequences.^27^^,^^28^^,^^29^ Specifically, individuals with EUG show self-control difficulties^30^ and impairment of cognitive functions, such as increased inattention and decreased verbal memory performance.^27^^,^^28^ In terms of inhibitory control, few studies have investigated the EUG impacts on inhibitory control behavior and neural rhythms. To our knowledge, only Luijten et al. reported reduced inhibitory control in the players with EUG, but the underlying EEG rhythms and ERP patterns have not been explored yet.^31^

Thus, the present study examined how EUG influences inhibitory control and related neural activities compared to non-gamers. We hypothesized that individuals with EUG would show impaired inhibitory control behavior. In addition, following the findings from IGD, we expected EUG players to have increased theta and alpha power in the resting state, especially in the frontal region. Furthermore, a larger P3 amplitude reflects more attention, better information processing, and better inhibitory functioning, while P3 latency is positively related to the time required for the evaluation or classification of stimuli.^26^^,^^32^ Consequently, we predicted reduced P3 amplitude or delayed P3 latency in EUG individuals.

## Results

### Behavioral performance

**Figure 1:**
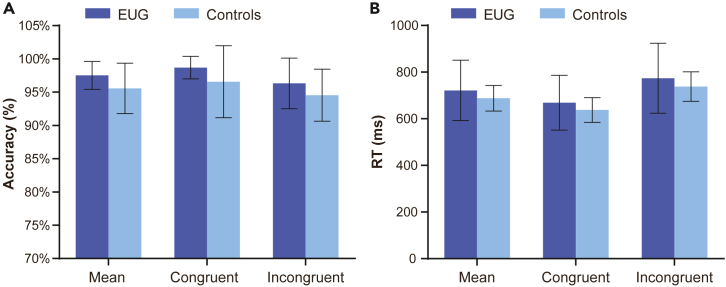
Behavioral performance of EUG group and the control group (A) Accuracy. (B) RT (error bar: SD).

For all detailed descriptive and statistical values, see Tables S1 and S2. Regarding the accuracy of the Stroop task, neither the main effect of the group nor the interaction between group and stimulus type was significant (*p* > 0.05), indicating that there was no significant difference between the two groups in accuracy (Figure 1A).

Similarly, the reaction time (RT) of the Stroop task did not show significance in either the main effect of the group or the interaction between group and stimulus type (*p* > 0.05), suggesting no significant difference between the two groups in RT (Figure 1B). In addition, the Stroop effect had no significant difference between the two groups [*F*(1, 27) = 0.009, *p* > 0.05, partial η^2^ = 0.000].

### ERP results

**Figure 2:**
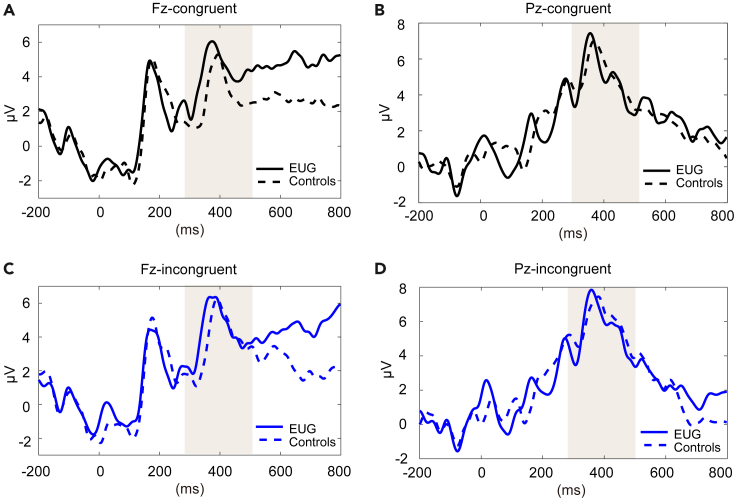
The grand average ERP waveforms of EUG group and the control group (A) Fz - congruent. (B) Pz - congruent. (C) Fz - incongruent. (D) Pz - incongruent.

The grand average ERP waveforms of the EUG group and non-gamers control group at Pz and Fz electrode sites with different stimulus types are shown in Figures 2A and 2B.

For the P3 amplitude at Pz and Fz electrode sites, neither the main effect of group nor the interaction between group and stimulus type showed significance (*p* > 0.05), suggesting that the two groups had no significant difference in P3 amplitude.

For the peak latency at Fz electrode site, the main effect of group was not significant (*p* > 0.05). However, a significant interaction effect [*F*(1, 26) = 4.880, *p* = 0.036, partial η^2^ = 0.158] was found. Further post-hoc analysis with Bonferroni correction revealed significantly shorter latency (*p* = 0.010) in the EUG group (M = 377.87, SD = 27.20) than non-gamers (M = 416.57, SD = 36.87) under incongruent condition. For the peak latency at Pz electrode site, neither the main effect of group nor the interaction between group and stimulus type showed significance (*p* > 0.05). For all detailed descriptive and statistical values, see Tables S3 and S4.

### Resting EEG results

**Figure 3:**
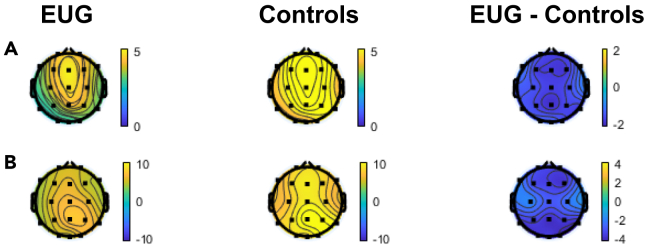
The EEG power topography of EUG group and the control group (A) Theta (4–8 Hz). (B) Alpha (8–12 Hz).

Regarding theta power, the group showed a significant main effect [group: *F*(1, 27) = 5.823, *p* = 0.023, partial η^2^ = 0.177], driven by significantly reduced (*p* = 0.023) theta power in EUG (M = 3.862, SD = 1.556) compared to non-gamers (M = 5.334, SD = 1.541), as presented in Figure 3A. However, the group × electrode site interaction was not significant (*p* > 0.05)**.** The above results suggested that the EUG group had lower theta power than non-gamers, which was independent of electrode location.

Alpha power revealed a significant main effect of group [group: *F*(1, 27) = 5.506, *p* = 0.027, partial η^2^ = 0.169], driven by significantly reduced alpha power (*p* = 0.027) in EUG (M = 5.681, SD = 3.01) compared to non-gamers (M = 8.691, SD = 3.341). Additionally, there was a significant group × electrode site interaction [*F*(2, 54) = 4.106, *p* = 0.022, partial η^2^ = 0.132]. The post-hoc test with Bonferroni correction showed that EUG had significantly reduced alpha power than non-gamers in frontal electrode site (EUG: M = 5.63, SD = 2.45; Control: M = 8.25, SD = 3.41; *p* = 0.023), and in parietal electrode site (EUG: M = 6.32, SD = 3.53; Control: M = 9.68, SD = 3.74; *p* = 0.017), while the alpha power in central electrode site had no significant difference between the two groups (*p* > 0.05), as shown in Figure 3B. For all detailed characteristics descriptions, see Tables S5 and S6.

## Discussion

Previous studies have found that the use of Internet games influences the inhibitory control and EEG activities, mainly for individuals with IGD. However, gaming effects on individuals with EUG are rarely known. Therefore, the present study investigated whether EUG significantly impacted inhibitory control and related neural activities compared to those not playing Internet games. The study found that no differences in behavioral indicators and P3 amplitude between individuals with EUG and non-gamers. However, EUG gamers exhibited shortened P3 latency, reduced theta and alpha power in the eyes-closed resting state.

EUG group exhibited significantly higher depression scores and anxiety scores compared to the control group, suggesting that excessive use of Internet games is associated with elevated levels of anxiety and depression. Our findings are in line with previous studies regarding the relationship between addiction and mental health outcomes.^33^^,^^34^ For instance, Wang et al. reported a positive association between mobile game addiction and social anxiety, depression, and loneliness.^34^ Similarly, Fayazi & Hasani concluded that Internet addiction, smartphone addiction, and online game addiction were all correlated with increased levels of social anxiety.^33^ These findings align closely with our own results, which indicate that individuals with EUG, even if they have not reached addiction-level tendencies, exhibit higher levels of depression and anxiety compared to non-users.

The color-word Stroop task is a tool for inhibitory control assessment, in which the incongruent stimuli require response inhibition to overcome increased cognitive interference.^16^^,^^17^^,^^35^^,^^36^^,^^37^ We found that the accuracy, RT, and Stroop effect were not significantly different between the two groups, suggesting that EUG did not influence the inhibitory control of individuals. This result differs from the reported impaired inhibition control in IGD population.^13^^,^^14^^,^^15^^,^^16^^,^^25^^,^^37^^,^^38^ For instance, Dong et al. reported impaired inhibitory control in individuals with IGD by fMRI Stroop task,^37^ go/no-go,^7^ and switching.^38^ Whereas for individuals with EUG who did not develop into IGD in this study, we did not find their reduced inhibitory control behavioral performances, which may be explained by the following two reasons. On the one hand, although both populations from IGD and EUG excessively play games, they are different subject populations. Comorbid disorders are common among the IGD population, including higher levels of psychopathology,^39^ anxiety disorders, depressive disorders,^40^ tolerance, withdrawal, conflict, and relapse.^41^ In contrast, EUG individuals are not addiction patients and do not have these symptoms. Another possible reason was that the Stroop task may not be sensitive enough to detect the changes in inhibitory control in EUG. Considering that some studies applied the event-related cue reactivity tasks in individuals with IGD, such paradigms related to game cues might be easier to assess the characteristics of inhibitory control in the gaming users.^39^^,^^40^^,^^41^^,^^42^^,^^43^

Regarding the P3 component, P3 amplitude reflects attentional resources employed in incongruent tasks, while P3 latency provides information about stimulus-factor effects and response processes.^44^ In addition, a larger P3 amplitude represents better inhibitory control ability.^45^ Previous correlational analysis revealed a significant negative correlation between P3 amplitude and the severity of IGD symptoms.^25^ Unlike IGD patients, we found that P3 amplitude in EUG individuals is comparable with non-gamers. It is reasonable since the EUG individuals who have not developed into addiction did show impaired inhibitory control in the Stroop task in this study. Such result is also different from problematic Facebook users, who showed less engagement in response inhibitory processes with lower Nogo-P3 amplitude to Facebook-related, pleasant, and neutral stimuli than to unpleasant stimuli, coincidentally opposite to nonproblematic Facebook users, who were activated successful inhibitory processes through Facebook-related and highly arousing emotional stimuli.^46^ We may attribute it to the difference between using social media and playing games, and further exploration is needed in future work.

Unlike P3 amplitude, the EUG group exhibited shorter P3 latency at Fz electrode site under incongruent conditions, which was opposite to our expectation. One possible reason is that the use of Internet games can improve users' visual short-term memory, perceptual skills, selective attention, cognitive flexibility, and working memory positively, while P3 latency is considered as an index of neural speed or brain efficiency.^47^^,^^48^^,^^49^^,^^50^ Thus, EUG individuals may have better cognitive functions especially inhibitory control reflected by shortened P3 latency.

Alpha-band oscillations play a regulatory role in many cognitive processes, such as perception, attention, working memory, and long-term memory.^51^^,^^52^^,^^53^ In addition, alpha appears more relevant to visual attention^54^ and visual-spatial working memory, especially in the frontal cortex.^53^ Researchers have reported that individuals with a decrease in alpha power of cortical regions have smaller behavioral switch costs, suggesting that alpha oscillations are critical to supporting cognitive flexibility.^55^ Studies using positron emission tomography (PET) and magnetic resonance imaging (MRI) in the thalamus and cortical areas showed that decreased alpha power is correlated with increased thalamic metabolism, reflecting higher activity or increased arousal.^56^^,^^57^ In this study, the decreased alpha power showed that when the individual had not developed into addiction, using Internet games might improve the individual’s inhibitory control, which still requires further examination in future work. In addition, we found that EUG individuals had shortened P3 latency and reduced resting alpha power, which is in line with the positive association between P3 latency and resting alpha activity in previous work.^50^^,^^58^^,^^59^^,^^60^ Both the shorter P3 latency and reduced alpha power might explain the less waste in attentional resources and more possibility in inhibitory control together.

It was found that individuals with EUG showed significantly reduced theta power under the eyes-closed resting state compared to non-gamers. Resting theta plays a vital role during response inhibition, such as processing and triggering different responses to congruent and incongruent stimuli in the central and integrating sensory and response-related processes in the frontal.^60^^,^^61^^,^^62^^,^^63^ Thus, we speculated that EUG with reduced theta may have higher inhibitory control.

### Limitations of the study

Several limitations of this study should be elucidated. First, there has been very little research on EUG, and the criterion of participant selection has not been standardized yet. Thus, our findings were limited to the participant selection criterion that was in line with previous work.^14^^,^^64^^,^^65^^,^^66^ Another possible limitation was that the sample size was relatively small, and the Stroop task may not be sensitive enough to assess inhibitory control in the EUG population. A larger sample size and the optimized experimental task would be desirable in future work. Finally, participants’ IQ scores or years of education should be considered as an important factor, which would be included in future research to provide a more comprehensive understanding of the relationship between gaming behavior and cognitive functioning.

### Conclusions

In conclusion, although EUG did not show differences in the behavioral measures of inhibitory control compared to non-gamers, their reduced alpha and theta power in resting state and shortened P3 latency together may imply that individuals with EUG performed better inhibitory control during the cognitive process. The results provide insight into the effects of Internet gaming on inhibitory control, which needs further confirmation with an appropriate experimental paradigm for inhibitory control assessment and a larger sample size in future work.

## STAR★Methods

### Key resources table

**Table:** 

REAGENT or RESOURCES	SOURCE	IDENTIFIER
**Software and algorithms**		

MATLAB 2021a	https://ww2.mathworks.cn/products/matlab.html	RRID: SCR_001622
Neuroscan SynAmps RT 64-channel Amplifier	https://compumedicsneuroscan.com/applications/eeg/	RRID: SCR_015818

### Resource availability

#### Lead contact

Further information and requests for resources should be directed to and will be fulfilled by the lead contact, Wenya Nan (wynan@shnu.edu.cn).

#### Materials availability

This study did not generate new unique reagents.

#### Data and code availability

• All data reported in this paper will be shared by the lead contact upon request.
• This paper does not report original code.
• Any additional information required to reanalyze the data reported in this paper is available from the lead contact upon request.

### Experimental model and study participant details

The participants were recruited from Shanghai Normal University through advertisements on the university, WeChat, and other online channels. In the period of experiment design, G∗power 3.1 software was used to calculate the required sample size. We set the effect size (*f*) to 0.25, the significance level (*α*) to 0.05, and the statistical power (1-*β*) to 0.80, which yielded a calculated sample size of 34 participants for the two groups. Excluding four subjects who left midway during the experiment, a total of 30 participants were included in the final statistical analysis.

Young’s Internet Addiction Test (IAT)^64^ was used to assess the severity of Internet gaming addiction. It consists of 20 items rated on a 5-point Likert scale (from 0 = 'never' to 4 = 'always'). The criteria for IGD in the DSM-5 is that a participant meets more than four items in a total of nine items: Persistent excessive use, tolerance, preoccupation, withdrawal, lack of other interests, functional impairment, deception of other people regarding game use, unsuccessful attempts to control Internet use and escape. The inclusion criteria of EUG participants were smartphone Internet game players, with IAT score from 50 to 80 and not meeting the IGD diagnostic criteria in DSM-5. The inclusion criteria for the control group were healthy adults who never played Internet games.^14^^,^^15^^,^^42^^,^^43^^,^^64^^,^^65^^,^^66^^,^^67^^,^^68^

Finally, the EUG group included 15 Chinese participants (11 males), and the control group consisted of 15 Chinese non-gamers (4 males). As shown in the following Table, the chi-square test on the sex ratio revealed a significant difference between the two groups (*χ*^2^ = 6.533, *p* = 0.011), while the independent sample *t*-test showed no significant difference in age between the two groups (*t* (28) = 0.78, *p* = 0.44). All participants were undergraduate and postgraduate students from Shanghai Normal University, healthy and with no psychiatric disorders history. The study was performed under the Declaration of Helsinki and approved by the Ethics committee of Shanghai Normal University. All participants signed written informed consent before the experiment started and got RMB 40 Yuan and a small gift for participation at the end of the experiment.

Considering previous research indicating severe levels of depression and anxiety among addiction populations, our study also assessed whether similar mental health challenges were present among individuals with EUG.^33^^,^^34^ The Beck depression inventory (BDI)^69^ and Beck anxiety inventory (BAI)^70^ were employed to measure the degree of depression and anxiety. Both tools consist of 21 self-report questions rated by the interviewees on a 0 ("never") to 3 ("very likely") scale. The independent sample *t*-tests on the IAT, BDI, and BAI scores showed significant differences between the two groups (see the **Table** below).

**Table:** Characteristic descriptions of Gender, Age, IAT, BDI, and BAI scores between the two groups.

Group	Gender (Male/Female)	Age (M ± SD)	IAT (M ± SD)	BDI (M ± SD)	BAI (M ± SD)
EUG	11/4	23.53 ± 1.68	64.20 ± 8.41	5.67 ± 2.61	24.80 ± 1.86
Control	4/11	23.06 ± 1.59	20.00 ± 0.00	3.07 ± 2.01	23.13 ± 1.30
Statistic	*χ*^2^ = 6.533^∗^	*t* = 0.78	*t* = 19.82^∗∗∗^	*t* = 3.01^∗∗^	*t* = 3.03^∗∗^

^∗^*p* < 0.05.

^∗∗^*p* < 0.01.

^∗∗∗^*p* < 0.001.

### Method details

#### EEG recordings

Participants were seated about 110 cm away from the computer screen and asked not to do any extra movement or blink during the experiment. They were required to relax under the eyes closed resting state for 4 min and then perform a Stroop task.

EEG recordings during the resting state and Stroop task were obtained from the scalp using an electrode Cap and a NeuroScan system (Scan 4.3; Compumedics Limited, Abbotsford, Australia). Twenty Ag-AgCl electrodes (FP1, FP2, F7, F3, FZ, F4, F8, T7, C3, CZ, C4, T8, P7, P3, PZ, P4, P8, O1, Oz, and O2) were mounted on an elastic cap and positioned according to International 10–20 system. The reference electrode was placed at the left mastoid, and the ground electrode was located between Pz and Fz. Moreover, the vertical electrooculogram (VEOG) was recorded by attaching additional electrodes above and below the left eye. The horizontal electrooculogram (HEOG) was recorded by attaching additional electrodes at the outer condyles of both eyes. All electrode impedances were kept below 5 kΩ. All signals were recorded at a sampling frequency of 500 Hz and a bandpass filter from 0.01–100 Hz.

#### Stroop task

We applied a color-word Stroop task to assess inhibitory control.^35^^,^^71^^,^^72^^,^^73^ E-prime 2.0 (Psychology Software Incorporation, Pittsburgh, Pennsylvania) was used to collect behavioral data. The task procedure is shown in the below Figure. First, the instruction was displayed on the computer screen. After the subjects understood the rule, they completed practice trials first. In the formal experiment, a white central fixation cross (+) would be presented at the center of the black screen for 250 ms, and then subjects needed to judge the ink of the word and make a reaction. After the reaction, the black screen would appear for 600–1400 ms, and the target word and black screen would appear for a total of 2000 ms. In this task, three target words (red, green, and yellow) would randomly present with congruent conditions (e.g., 'red' with red ink) or incongruent conditions (e.g., 'red' with green or yellow ink). The subjects were required to press a button according to the ink of the word. If the ink was red, they should press 'F'. If the ink was green, they should press the space key, and if the ink was yellow, they should press 'J'. Each participant performed one block of 144 trials, for which 72 congruent and 72 incongruent trials were presented randomly.

**Figure:**
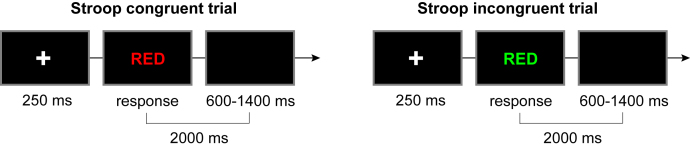

#### Resting EEG offline analysis

The EEG signals were processed by customized scripts and EEGLAB toolbox^74^ in MATLAB software. The signals were re-referenced to the mean of both mastoids and bandpass filtered from 0.5–45 Hz. Then the signals were segmented into 4-s epochs. Subsequently, the electrooculogram (EOG) and electromyogram (EMG) artifacts were corrected automatically through the blind source separation (BSS)-based EOG correction^75^ and canonical correlation analyses (CCA) correction algorithms,^76^ respectively, available in the automatic artifact removal (AAR) plug-in.^76^ Finally, the epochs with absolute amplitude above 70 μV were rejected.

The fast Fourier transform (FFT) was used to calculate the EEG power at each electrode in the theta (4–8 Hz) and alpha (8–12 Hz) frequency bands. In addition, twenty electrodes were divided into the frontal (FP1, FP2, F7, F3, FZ, F4, and F8), central (T7, C3, CZ, C4, and T8), and parietal-occipital positions (P7, P3, PZ, P4, P8, O1, OZ, and O2), and the average power of all electrodes in each electrode position was calculated respectively.

#### ERP analysis

The EEG signals during the Stroop task were first down-sampled into 250 Hz. Data were then re-referenced to the mean of both mastoids and filtered from 0.1 to 30 Hz. Eye blinks and muscle artifacts were identified and removed using independent component analysis (ICA), then we used ICLabel to classify and reject artifacts automatically. Data were then segmented into epochs of 1000 ms, which included the 200-ms pre-stimulus baseline period. Epochs with absolute voltage over 70 μV were discarded automatically. One participant’s EEG data from the EUG group did not have event markers during EEG recordings, thus was excluded from ERP analysis. Consequently, the ERP analysis in the EUG group included 14 participants. Only trials with correct responses were averaged and analyzed. The independent sample *t*-test revealed that the two groups had no significant differences in the number of correct trials of the P3 component for the congruent condition (EUG: 71 ± 1.254; Control: 69.53 ± 3.871; *t*(28) = 1.396, *p* = 0.181), and for the incongruent condition (EUG: 69.40 ± 2.746; Control: 68.07 ± 2.815; *t*(28) = 1.313, *p* = 0.200). According to previous studies, the P3 component includes two subcomponents, P3a and P3b.^77^^,^^78^^,^^79^ Specifically, P3a refers to the frontal (maximum at Fz) activity and P3b refers to the parietal (maximum at Pz) activity. Thus, we assessed the P3 activity by the local peak latency and local peak amplitude within 300–500 ms post-stimulus for Fz and Pz (following the International 10–20 system). The peak amplitude and latency of P3 for each subject were calculated with ERPLAB.

### Quantification and statistical analysis

In order to control the impact of gender differences between the two groups, we conducted ANOVA with gender as a covariate in the following analysis.

The accuracy and RT were taken as the performance measures of Stroop task. For each performance measure, repeated-measures ANOVA was performed with group (2 levels: EUG, Control) as the between-subject factor and stimulus type (2 levels: congruent, incongruent) as the within-subject factor and gender as the covariate. Furthermore, we performed ANOVA with gender as the covariate for the Stroop effect (i.e., the RT in the incongruent condition minus the RT in the congruent condition).^73^^,^^80^

For the P3a and P3b amplitude and latency, repeated-measures ANOVA was performed with group (2 levels: EUG, Control) as the between-subject factor and stimulus type (2 levels: congruent, incongruent) as the within-subject factor and gender as the covariate.

For the resting-state EEG power, ANCOVA was performed with group (2 levels: EUG, Control) as the between-subject factor and electrode site (3 levels: frontal, central, parietal-occipital) as the within-subject factor and gender as the covariate.

The above statistical analyses were conducted using SPSS 23 Software (SPSS Inc., Chicago, IL, USA). The significance level was set at 0.05 (*∗* represents *p* < 0.05, *∗∗* represents *p* < 0.01, *∗∗∗* represents *p* < 0.001). The Greenhouse-Geisser correction was applied once the violation of spherical hypothesis. Once the main effect or the interaction was significant, post-hoc comparison with the Bonferroni correction was applied.

### Additional resources

This paper did not create any additional resources.
