# Supplementary material for: Effect of excessive internet gaming on inhibitory control based on resting EEG and ERP
Source: iScience. 2024 Jun 27;27(8):110399. doi: 10.1016/j.isci.2024.110399 (PMC11284701; doi:10.1016/j.isci.2024.110399)
Supplement: Document S1. Tables S1–S6 [file mmc1.pdf]

iScience, Volume *27*

Supplemental information

Effect of excessive internet gaming on inhibitory control based on resting EEG and ERP

Jiayi Xu, Lu Shen, Huajia Fei, Wenbin Zhou, Feng Wan, and Wenya Nan

Supplementary Materials

Table S1. The RT and Accuracy of EUG and control groups (Mean ± SD), related to Figure 1.

	RT (ms)		Accuracy (%)	
	Congruent	Incongruent	Congruent	Incongruent
EUG	674.9±117.5	783.7±150.0	98.6±1.7	96.3±3.8
Control	637.8±53.0	737.8±63.6	96.5±5.4	94.5±3.9

Table S2. Behavior comparisons using repeated-measures ANOVA with gender as a covariate, related to Figure 1.

Variable	Factor	F	p	η_p^2
RT (ms)	Group	0.356	0.556	0.013
	Group*Stimulus type	0.009	0.927	0.000
Accuracy (%)	Group	2.095	0.159	0.072
	Group*Stimulus type	0.117	0.735	0.004

Table S3. The amplitude and latency of P3 with different stimulus types in EUG and control groups (Mean ± SD), related to Figure 2.

		Amplitude (μV)		Latency (ms)	
		Congruent	Incongruent	Congruent	Incongruent
P3a	EUG	1.72±2.21	1.85±1.82	401.33±50.23	384.40±41.56
	Control	1.32±1.65	1.67±2.40	398.71±34.70	377.14±29.22
P3b	EUG	2.71±1.35	3.23±0.86	377.87±27.20	388.53±47.49
	Control	2.66±1.18	3.34±1.60	416.57±36.87	378.71±27.54

Table S4. The amplitude and latency of P3 comparisons using repeated-measures ANOVA with gender as a covariate, related to Figure 2.

Variable		Factor	F	p	η_p^2
Amplitude (μV)	P3a	Group	0.978	0.332	0.036
		Group*stimulus type	0.016	0.899	0.001
	P3b	Group	0.000	0.999	0.000
		Group*stimulus type	0.008	0.930	0.000
Latency (ms)	P3a	Group	1.528	0.228	0.055
		Group*stimulus type	4.880	0.036*	0.158
	P3b	Group	0.934	0.343	0.035
		Group*stimulus type	0.000	0.987	0.000

Table S5. The theta and alpha power at frontal, central, and parietal electrode sites of EUG and control groups (Mean ± SD), related to Figure 3.

		Frontal	Central	Parietal
Theta power	EUG	4.47±1.46	3.97±1.64	3.37±1.45
	Control	5.79±1.55	5.18±1.37	4.82±1.40
Alpha power	EUG	5.63±2.45	5.75±3.13	6.32±3.53
	Control	8.25±3.41	7.49±2.61	9.68±3.74

Table S6. The resting EEG comparisons using repeated-measures ANOVA with gender as a covariate, related to Figure 3.

Variable	Factor	F	p	η_p^2
Theta power	Group	5.823	0.023*	0.177
	Group*Electrode site	0.309	0.736	0.011
Alpha power	Group	5.506	0.027*	0.169
	Group*Electrode site	4.106	0.022*	0.132
